# Hippo Signaling Pathway as a New Potential Target in Non-Melanoma Skin Cancers: A Narrative Review

**DOI:** 10.3390/life11070680

**Published:** 2021-07-12

**Authors:** Igor Aleksander Bednarski, Magdalena Ciążyńska, Karolina Wódz, Izabela Dróżdż, Małgorzata Skibińska, Joanna Narbutt, Aleksandra Lesiak

**Affiliations:** 1 Department of Dermatology, Pediatric Dermatology and Dermatological Oncology, Medical University of Lodz, 91-347 Lodz, Poland; malgorzata.skibinska@umed.lodz.pl (M.S.); joanna.narbutt@umed.lodz.pl (J.N.); lesiak_ola@interia.pl (A.L.); 2 Department of Proliferative Diseases, Nicolaus Copernicus Multidisciplinary Centre for Oncology and Traumatology, 93-513 Lodz, Poland; magdalena.ciazynska@umed.lodz.pl; 3 Laboratory of Molecular Biology, VET-LAB Brudzew, 62-720 Brudzew, Poland; karolina.wodz@labbrudzew.pl; 4 Department of Clinical Genetics, Medical University of Lodz, Pomorska 251, 92-213 Lodz, Poland; izabela.drozdz@umed.lodz.pl

**Keywords:** Hippo signaling, basal cell carcinoma, squamous cell carcinoma, YAP

## Abstract

Non-melanoma skin cancers (NMSCs), including basal cell carcinoma (BCC) and cutaneous squamous cell carcinoma (cSCC), are the most frequently diagnosed cancers in humans, however, their exact pathogenesis is not fully understood. In recent years, it has been hypothesized that the recently discovered Hippo pathway could play a detrimental role in cutaneous carcinogenesis, but no direct connections have been made. The Hippo pathway and its effector, YAP, are responsible for tissue growth by accelerating cell proliferation, however, YAP upregulation and overexpression have also been reported in numerous types of tumors. There is also evidence that disrupted YAP/Hippo signaling is responsible for cancer growth, invasion, and metastasis. In this short review, we will explore whether the Hippo pathway is an important regulator of skin carcinogenesis and if it could be a promising target for future therapies.

## 1. Introduction

Non-melanoma skin cancers (NMSCs), including basal cell carcinoma (BCC) and cutaneous squamous cell carcinoma (cSCC), are the most frequently diagnosed cancers in humans [1]. Despite having a relatively good prognosis, NMSC represents an important public health concern due to its high prevalence and cost of treatment. BCC, comprising 70% of NMSCs, has a low degree of malignancy and has limited metastasis potential, however, in some cases it shows the invasion and destruction of surrounding tissues [1,2]. Cutaneous SCC presents a much more aggressive phenotype with a high risk of local recurrence and metastatic ability [1]. Current research focuses on finding pathways/genes involved in cutaneous carcinogenesis and, in recent years, a new signaling pathway named the Hippo pathway, has been proposed as a novel approach to NMSC development.

Gene expression profiling in *Drosophila melanogaster* led to the discovery of a new signaling pathway, Salvador/Warts/Hippo, that determines tissue growth by promoting cell proliferation and inhibiting apoptosis [3]. It was later demonstrated that the pathway is highly conserved among mammals, and in many recent studies it has been hypothesized that the transcriptional co-activator YAP (Yes-associated protein), the Hippo pathway effector, could be one of the key regulators of cutaneous carcinogenesis, and thus appears to be a promising site of action for targeted therapies [4,5].

It has been shown that the activity of the YAP and its biological role is regulated by its phosphorylation; depending on the site of phosphorylation it can both induce and protect against apoptosis. This is particularly important in tumors where the resistance of tumor cells to apoptosis can determine the success of the treatment and the survival time. Although it is well known that various signaling pathways are involved in the tissue growth process, it is still not clear how organ size information, which is governed partially by the Hippo pathway, is integrated and translated into cell proliferation and apoptosis [6,7].

Due to the proliferative and anti-apoptotic properties of the YAP, many studies focused on the role of this protein in carcinogenesis. The deregulation of the YAP expression has been reported in a broad range of different human cancers, including non-small cell lung carcinoma (nSCLC) [8], ovarian [9], hepatocellular carcinoma (HCC) [10], pancreatic cancer [11], and malignant melanoma [12]. The foregoing studies on the YAP relied on the immunohistochemical staining of the YAP in the cell nucleus of tumor tissue, as the YAP induces a growth-promoting transcriptional programme when it is translocated into the nucleus [13]. An abundance of the nuclear YAP was found in approximately 60% of HCC [10], 15% of ovarian cancers [14], and 65% of nSCLC [8]. Furthermore, the overexpression of the YAP in the MCF10A cell line (epithelial cell line taken from the mammary gland) may induce the epithelial–mesenchymal transition (EMT) and protect cells from apoptosis [4]. It has also been suggested that the YAP may act as an oncogene due to the activation of genes responsible for cell proliferation, chemoresistance, metastasis, and the acquisition of cancer stem cell properties [5].

In this short review, we would like to explore deregulation within the Hippo pathway in NMSCs and to discuss the possible implications of targeting the Hippo pathway in NMSC treatment.

## 2. Hippo Pathway Signaling Cascade

**Figure 1:**
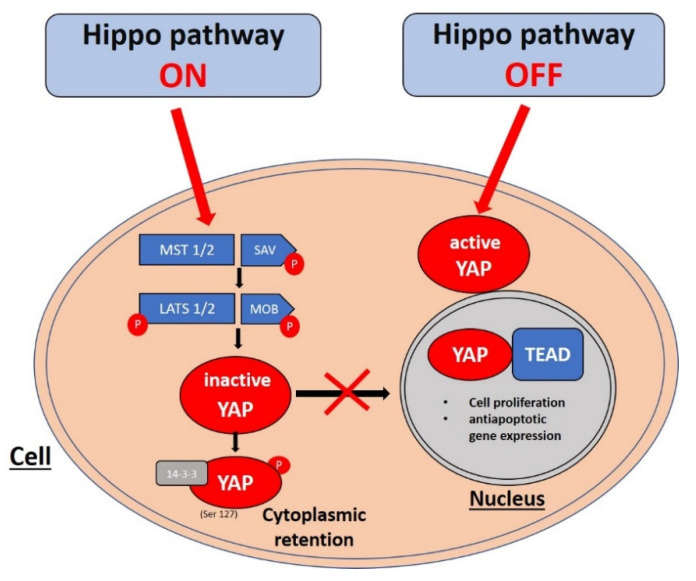
Hippo signaling pathway cascade relies on the activation of its downstream target, YAP. Phosphorylation cascade mediated via MST1/2 and LATS1/2 results in YAP phosphorylation and cytoplasmic retention thus preventing its nuclear translocation. When the Hippo pathway is “off”, the YAP is transferred into the cell nucleus and binds with the TEAD starting the transcription of antiapoptotic and survival-promoting genes.

The Hippo pathway forms a phosphorylation cascade consisting of MST1 and 2 (mammalian Ste20-like kinases 1 and 2), LATS1 and 2 (large tumor suppressor 1 and 2), their cofactors SAV1 (Salvador family WW domain-containing protein 1) and MOB1 (MOB kinase activator 1), and the pathway effector YAP (7). MST1 and MST2 kinases and their cofactor SAV1 form the complex which phosphorylates LATS1 and LATS2 kinases [7]. Activation of LATS1/2 kinases results in the phosphorylation of the YAP in S127 serine residue. This phosphorylation inhibits YAP activity by binding it to the 14–3–3 protein, thus preventing it from translocating to the cell nucleus [15]. The lack of phosphorylation of the YAP in S127 residue results in its migration to the nucleus, however, the YAP, as a transcriptional coactivator, is unable to bind to DNA. Therefore, in order to stimulate the expression of target genes responsible for cell proliferation and antiapoptotic action, it binds with transcriptional factors of the TEAD (transcriptional enhancer factor domain) family, which can directly bind to DNA [16]. Genome-wide analyses of YAP transcriptional targets revealed CTGF (connective tissue growth factor) and ANKRD1 (ankyrin repeat domain 1) as the main YAP target genes [17,18]. However, the YAP does not work exclusively as an apoptosis inhibitor. It has been demonstrated that in UV-induced DNA damage, the YAP could also act as the proapoptotic factor by binding to the p73 transcriptional factor (which belongs to the p53 family). Binding the YAP to p73 protects p73 against proteasomal degradation by E3 ligase Itch [19]. The Hippo signaling pathway cascade is shown in Figure 1.

The growing evidence indicates that these opposing roles played by the YAP are dependent on its sites of phosphorylation. YAP phosphorylation is a key mechanism that determines its biological activity and cell location and may occur in many places. One of the most well-known phosphorylation mechanisms occurs in S127 serine residue by LATS1/2. The YAP may also be phosphorylated by other kinases, including JNK1/2, ERK2, PKCα, and c-Abl [17,20,21]. YAP phosphorylation by c-Abl is a clear example that the role of the YAP is regulated by its phosphorylation. DNA damage causes the c-Abl kinase to phosphorylate the YAP on tyrosine residue 357, which binds to p73 and promotes transcription of proapoptotic gene promoters, including BAX [21]. However, Tomlinson et al. [22] found that during the exposure of keratinocytes to UVC, JNK1/2 kinases phosphorylate YAP in five previously undescribed sites: T119, S138, T154, S317, and T362. The YAP phosphorylation by JNK1/2 in keratinocytes protects them from apoptosis by stabilizing p63 (which also belongs to the p53 protein family), but in squamous cell carcinoma cells, it stimulates apoptosis by binding to p73. Studies conducted by Lee et al. [20] confirmed these findings and showed that YAP activity, after exposure to genotoxic stress (UV radiation, cisplatin), is regulated by hyperphosphorylation.

## 3. Basal Cell Carcinoma

Sporadic basal cell carcinoma (BCC) is one of the most common human cancers with increasing incidence worldwide [23]. So far, extensive studies have confirmed that BCC development is orchestrated by mutations in the Sonic Hedgehog pathway regulators, especially in the Patched 1 receptor (*PTCH1*) and G-protein-coupled receptor Smoothened (*SMO*) which eventually results in the activation of nuclear, GLI-dependent growth programs [24]. A mechanistic molecular approach to the Hedgehog pathway has led to the discovery of vismodegib, an antagonist of SMO, however, it was later discovered that infiltrative and metastatic BCC could avoid the SMO inhibition which raised suspicions that other signaling pathways may be involved in this process [25,26]. Genomic analyses revealed that loss-of-function mutations within *PTPN14* and *LATS1* resulted in the accumulation of YAP in the nucleus [24,27], suggesting the involvement of the Hippo pathway in the development of BCC.

Nuclear YAP expression seems to be one of the hallmarks of BCC. Quan et al. [28] reported that in normal adult human skin the YAP is predominantly expressed within the cytoplasm of epidermal keratinocytes in the interfollicular epidermis and outer sheath of the hair follicles, while, in BCC, the expression of the YAP was found in the tumor island cells both in the cytoplasm and nuclei with 10-fold greater protein levels in BCC tumor islands than in normal epidermis [28] indicating atypical YAP regulation in BCC. Debaugnies et al. [29] showed, on murine models, that the deletion of *Ptch1* or constitutive activation of *Smo* resulted in BCC development with nuclear YAP expression [29]. To extrapolate those findings to a human model, they analyzed superficial, nodular, and infiltrative types of human BCC using immunostaining and provided evidence that, regardless of any cancer-initiating event (oncogene activation, tumor suppressor mutation) or BCC histological subtype, nuclear YAP expression takes place [29]. These findings were further supported by Maglic et al. [30] who confirmed that intranuclear YAP location is one of the histological signs of both human and murine BCC [30].

Despite growing evidence that the YAP participates in BCC development there is still little known about the possible implications of YAP activation. Cysteine-rich protein 61 (CCN1) and connective tissue growth factor (CCN2, also known as CTGF) are directly targeted by YAP transcription [31,32] with different cellular functions e.g., extracellular matrix production, apoptosis, and cell proliferation [33]. Since their role in basal cell carcinoma is poorly understood, Quan et al. [28], in a series of experiments, showed that the YAP upregulates *CCN1* and *CCN2* in BCC tumor cells which results in elevated CCN1 and CCN2 mRNA levels, and modulates healthy keratinocyte proliferation via CCN1 [28]. Knocking down YAP in the human keratinocyte cell line displayed reduced expression of CCN1 and CCN2 as well as the loss of keratinocyte proliferation [28]. Interestingly, after the restoration of CCN1 and CCN2 expression, it was discovered that CCN1 restored keratinocyte proliferation [28]. On the contrary, the knockdown of CCN1 but not CCN2 in YAP-expressing keratinocytes also reduced the keratinocyte proliferation exposing the critical role of the YAP in cell survival [28]. The essential role of the YAP in this context has been also exposed by Debaugnies et al. who showed that YAP knockout combined with SMO activation results in an increased apoptotic rate of epidermal cells compared to SMO-expressing cells without YAP knockout, indicating that YAP expression is also required for the survival of oncogene-expressing cells [29].

Considering that the Hedgehog pathway is pivotal for BCC development it becomes important to determine whether there is an existing connection between the Hedgehog and Hippo pathways in diseased skin. Medulloblastoma, the most common solid cancer in children, arises in the developing central nervous system from cerebellar granule neuron precursor (CGNP) cells in which proliferation is mediated via the disrupted activation of the Hedgehog pathway [34]. Current data suggest that Hedgehog signaling promotes YAP accumulation and nuclear localization in proliferating CGNPs, while YAP knockdown efficaciously reduces the proliferation rate of CGNP suggesting possible Hippo pathway involvement in Hedgehog-mediated proliferation [35]. A study by Akladios et al. [36] revealed several regulatory interactions between the Hippo and Hedgehog signaling pathways in BCC. Using a murine model with constitutional Smoothened mutant protein (SmoM2) expression (K14-CreER/Rosa-SmoM2 mice) that resulted in BCC development, they discovered increased Hedgehog signaling activity with YAP and GLI2 (a downstream target of Hedgehog signaling in the skin) nuclear expression accompanied by the increased expression of certain YAP target genes including *Ctgf* [36]. Moreover, using transgenic mice with YAP mutant protein activation (YAP2-5SA-ΔC mice) revealed the accumulation of GLI2 in the nucleus which was driven by β-catenin activation, and this suggested possible positive regulatory feedback between the Hippo and Hedgehog pathways in the pathogenesis of BCC [36]. Furthermore, Debaugnies et al. [29] found that constitutive SMO activation without YAP knockout in mice resulted in the development of BCC while in mice with YAP knockout, no such event occurred indicating that YAP activation seems to be crucial in the development of SMO-dependent BCC [29]. Interestingly, in the last part of their experiment, Debaugnies et al. also showed YAP knockout combined with SMO activation resulted in an increased apoptotic rate of epidermal cells compared to SMO-expressing cells without YAP knockout implicating that YAP expression is also essential for the survival of oncogene-expressing cells [29]. The essential role of the YAP in BCC progression has been endorsed by Maglic et al. [30], who reported that *Yap^+/+^* mice develop BCC after 8 weeks of tamoxifen administration, while in *Yap^fl^*^/*fl*^ mice only after 22 weeks, confirming the critical role of the YAP in BCC progression [30].

Basal cell carcinoma progression is not only driven by YAP overexpression. Tate et al. [37] reported the case of a female patient with Gorlin syndrome (also known as nevoid basal cell carcinoma syndrome, NBCCS) who developed multiple superficial and infiltrative basal cell carcinomas on her face and scalp [37]. Using whole-genome sequencing (WGS) analysis, they found a nonsense mutation in the *LATS1* gene (c. 943 C > T) and loss of the wild-type allele of *LATS1* indicating biallelic disruption within *LATS1.* Even more important, they compared the *LATS1* sequences between the superficial and infiltrative BCC and found no nucleotide alterations in the superficial BCC which showed the involvement of the Hippo pathway in BCC progression, highlighting another possible therapeutic target [24,37,38].

## 4. Squamous Cell Carcinoma

Cutaneous squamous cell carcinoma (cSCC) is the second most common non-melanoma skin cancer and accounts for approximately 20% of skin cancers [39,40]. In contrast to BCC, cSCC can metastasize, has an increased risk of local recurrence, and a higher mortality rate [39], therefore understanding its molecular hallmarks is imperative. Risk factors for cSCC include Fitzpatrick skin phototype I-III, excessive exposure to UV radiation, immunosuppression, and exposure to chemical/physical agents such as nitrosoamines, arsenium, and ionizing radiation [40]. It has been described that the development of cSCC is orchestrated via a complex interplay between several genes including *TP53*, *NOTCH1*, *CDKN2A*, *EGFR*, and RAS/RAF/MEK/ERK and PI3K/AKT/mTOR signaling pathways [41], however, in recent years several studies have discovered that the Hippo pathway could also play a detrimental role in this process. Debaugnies et al. [29] and Sambandam et al. [42] demonstrated, on a murine model, that the YAP is necessary for SCC cell initiation and cell proliferation, while genome-wide screening revealed that the YAP drives clonal cell expansion by the TEAD-dependent transcription of survival-promoting genes in both healthy skin and cSCC [43], which suggests the vital role of the YAP/Hippo pathway in cSCC.

Cutaneous squamous cell carcinoma can arise de novo or from precancerous skin lesions including actinic keratosis (AK) and Bowen’s disease (BD) [44]. Typically, cSCC formation starts with UV-induced damage within the interfollicular epidermis eventually leading to actinic keratosis or Bowen’s disease [45]. According to current research, the risk of transformation into cSCC is estimated between 3–5% for Bowen’s disease [46] and 0.1–10% for actinic keratosis [47].

Since the Hippo signaling pathway controls both epidermal homeostasis and keratinocyte proliferation, it is assumed that the Hippo pathway could also be dysregulated in cSCC as well as in BD and AK. Jia et al. [48] showed that the YAP is expressed within cells, parakeratotic columns in AK (45.2% of YAP-positive staining), and atypical epidermal cells (70.6% of YAP-positive staining) in BD indicating the possible Hippo pathway involvement in precancerous lesion formation [48]. Activation of the YAP in AK/BD was later confirmed by Al-Busani et al. [49] who reported nuclear YAP localization in 82.1% of cases of BD and 66.7% of cases of AK [49]. Moreover, YAP expression was also found in cSCC, in both well and poorly/moderately differentiated tumors, showing the expression of the YAP is upregulated in all stages of cSCC development [48,49]. Although YAP/Hippo signaling is disturbed in cSCC it is still not clear what the implications of YAP up-/downregulation are. Schlegelmilch et al. [50] demonstrated that epidermal YAP overexpression in transgenic mice led to the development of tumors morphologically resembling human cSCC [50]. After invading the derma, the tumors displayed signs of epithelial–mesenchymal transition which is also typical for advanced human cSCC [50]. In contrast, the downregulation of the YAP inhibits cSCC growth in vivo [48].

Considering that PI3K/AKT/mTOR and RAS/RAF/MEK/ERK pathways are major signaling pathways involved in cSCC development, it becomes important to determine a connection between them and YAP/Hippo signaling. Jia et al. [48] showed that the YAP activates both pathways via the regulation of RAS, indicating that YAP-dependent cSCC proliferation is indirectly maintained by RAS/ERK and PI3K/AKT signaling [48]. Moreover, YAP overexpression protected cells against apoptosis induced by 5-fluorouracil (commonly used topical treatment in cSCC) [48]. In contrast, studies on a YAP^flox/flox^ double-knockout murine model revealed that YAP deficiency prevents cSCC formation driven by AP-1/K-Ras [29,51].

While the incidence of metastatic BCC varies from 0.0028% to 0.55% [52], cSCC does metastasize in approximately 4% of cases with a mortality rate of >70% [53] resulting in a significant healthcare burden. Due to their poor prognosis and high frequency, it is of vital importance to understand the factors involved in the metastatic process. So far, there is not enough evidence to conclusively determine that the YAP plays a role in the cSCC metastatic process. Spindle cell carcinoma (spSCC) is a rare, aggressive subtype of cSCC that usually arises on radio- or photoexposed areas [54] due to epithelial–mesenchymal transition. The clinical risk associated with spSCC has not been quantified yet but it is suspected that its metastatic ability is higher than in cSCC [54]. EMT, characterized by the downregulation of E-cadherin and upregulation of vimentin, is associated with an increased risk of cSCC metastasis [55,56]. It has been revealed that cSCC transformation into spSCC is YAP-dependent and both the YAP and vimentin are highly expressed in human spSCC [56]. Remodeling of the extracellular matrix is another vital component of tumor formation allowing its metastatic invasion [57]. Although it has been revealed that silencing the YAP decreases the invasive ability of cSCC cells and results in decreased expression matrix metalloproteinases involved in tumor metastasis, e.g., MMP-2 and MMP-9 [48], it has still not been elucidated how YAP/Hippo signaling controls ECM homeostasis in cSCC. In Table 1 we present the mutation frequency in Hippo pathway genes for basal cell carcinoma and cutaneous squamous cell carcinoma.

## 5. YAP/Hippo Signaling as a Therapeutic Target in NMSC

There are no established treatments targeting YAP/Hippo signaling which are used in clinical practice, however, there are some compounds that are under investigation [59,60]. Still, the discovery of therapy that regulates the Hippo signaling pathway could be the next milestone in cancer therapy. Overexpression of the YAP has been confirmed in numerous types of cancer and is associated with excessive cell proliferation, evasion of apoptosis, and tissue invasion, so targeting the YAP, its translocation to the cell nucleus and YAP-TEAD complexes, seems to be a reasonable therapeutic approach in treating tumors with YAP hyperactivation [8,9,10,11,12]. The foregoing studies revealed that YAP–TEAD interaction could be disrupted by using particular drugs used in clinical practice including verteporfin, flufenamic acid, statins, and bisphosphonates [61,62,63].

Verteporfin is a photosensitizer used in the photodynamic therapy of age-related macular degeneration (AMD) and exhibits the ability to specifically bind the YAP and showed it could inhibit YAP-induced hepatomegaly [61]. However, in a murine melanoma model, verteporfin administration did not result in the disruption of melanoma initiation or progression [64].

Flufenamic acid, one of the non-steroid anti-inflammatory drugs (NSAID), has been shown to have high affinity in binding to the TEAD hydrophobic central pocket. While not having been tested in the treatment of cancer, flufenamic acid suppresses YAP-dependent transcription, cell proliferation and migration, therefore targeting the TEAD central pocket seems to be another solution in the treatment of tumors with dysregulated YAP/Hippo signaling [62,65]. Interestingly, the TEAD Tondu (TDU) domain could also serve as a viable therapeutic target. Vestigial-like family member 4 (VGLL-4) is a transcriptional regulator that pairs with TEADs via TDU domains [66]. By binding TEADs, VGLL-4 competitively restricts YAP–TEAD interaction and eventually inhibits targets downstream of the Hippo pathway [66]. These findings resulted in the development of a VGLL-4-mimicking peptide that inhibits in vitro growth of human primary gastric cancer [67].

Recent research demonstrated that YAP activity is also modulated via the mevalonate pathway by suppressing YAP phosphorylation using Rho-GTPases independently of LATS1/2 kinases, resulting in the nuclear translocation of the YAP [63]. Mevalonate pathway inhibitors zoledronic acid, along with HMG-CoA (3-hydroxy-3-methyl-glutaryl-coenzyme A) reductase inhibitors (cerivastatin and simvastatin), have been shown to impair YAP-dependent transcription in human breast adenocarcinoma cells [63]. Interestingly, there is currently a clinical trial in its second phase that uses the combination of zoledronic acid and atorvastatin in triple-negative breast cancer to assess the complete pathological response rate in comparison to standard neoadjuvant therapy, however, the results have not been published yet (NCT03358017) [68]. In Table 2 we summarize therapeutic approaches that regulate YAP/Hippo signaling in regards to cancer treatment.

## 6. Conclusions

In this review of Hippo pathway targeting and its role in the development and treatment of NMSC, only some of the many aspects could be highlighted. There is much evidence showing that the Hippo pathway is involved in the development of NMSC, yet more research is needed to fully understand its impact on skin carcinogenesis. Current research has proven that the Hippo pathway stimulates cell proliferation, modulates NMSC’s metastatic and invasive abilities, and helps tumor cells evade apoptosis.

Despite extensive research, there are still many unanswered questions, including the role of ultraviolet radiation in NMSC promotion and progression through the Hippo pathway, and mechanisms of drug resistance. Since NMSCs are the most common cancers in the human population, it is our belief that an understanding of the Hippo pathway may be vital in the development of clinically relevant targeted therapies, to predict the course of the disease and the prevention of NMSC, however, more studies are urgently needed.

## Figures and Tables

**Table 1:** Mutation frequency in main components of the Hippo signaling pathway (according to studies registered at http://www.cbioportal.org/, accessed on 5 July 2021) [58].

Gene Name	Mutation Frequency (%)	
	BCC (*n* = 293)	cSCC (*n* = 151)
*YAP*	1.7	2.6
*TEAD1*	0.7	0.7
*TEAD2*	1.4	7.3
*TEAD3*	-	2.0
*TEAD4*	0.3	2.6
*LATS1*	8.5	11.9
*LATS2*	9.2	11.3
*MST1*	1.7	3.3
*MST2*	-	-

**Table 2:** Potential cancer therapeutic approaches targeting YAP.

Compound	Mechanism of Action	Clinical Relevance	References
Verteporfin	Directly binds YAP	No effect on melanoma initiation/progression	[61,64]
Flufenamic acid	Disrupts YAP–TEAD interaction	None reported	[62]
VGLL-4-mimicking peptide	Disrupts YAP–TEAD interaction	Inhibits in vitro growth of primary human gastric cancer	[66]
Zoledronic acid	Inhibits YAP phosphorylation	In clinical trial in triple-negative breast cancer	[63,68]
Statins	Inhibits YAP phosphorylation		

## Data Availability

Not applicable.
